# Chronological lifespan in stationary culture: from yeast to human cells

**DOI:** 10.18632/aging.100406

**Published:** 2011-12-10

**Authors:** Zoya N. Demidenko

**Affiliations:** Oncotarget Inc., Buffalo, NY

A decade ago, Mikhail Blagosklonny predicted that cellular senescence is driven by mitogenic pathways, when the cell cycle is blocked and actual growth is impossible [1]. In particular, the mitogen- and nutrient-sensing mTOR (Target of Rapamycin) pathway drives either cell mass growth or aging [2]. Rapamycin prevents conversion of reversible cell cycle arrest to senescence [3-11]. When the cell cycle is blocked, but mTOR is still active, then cells become senescent. This process was named *gerogenic conversion* or simply *geroconversion* [12]. Rapamycin did not by-pass arrest but suppress geroconversion. Cells remained resting but not senescent. The discovery of mTOR-dependent geroconversion allowed Blagosklonny to connect cellular aging to age-related diseases and organismal aging [13, 14]. Furthermore, this predicted that rapamycin is a gerosuppressant, which could be used to prevent age-related diseases by slowing down the aging process [14]. Independently, it was discovered that rapamycin suppresses chronological aging of yeast cells [15]. Chronological lifespan of yeast cells in stationary culture is the most fruitful model in aging research and dozens of papers have been published in *Nature*, *Science*, *Cell* and *Cell Cycle*. Although yeast model was so useful to identify genes involved in mammalian aging, the reason remained unclear. Yeast only loosely resemble post-mitotic cells in human tissues. Unfortunately, there was no model of mammalian chronological aging in cell culture. In one model developed by Fabrizio and Valter Longo [16] as well as by Matt Kaeberlein and Brian K. Kennedy [17], yeast chronological senescence (CS) is caused by acidosis due to overproduction of acetic acid. Obviously, neither replicative nor accelerated senescence of human cells resembles yeast CS in the stationary culture. Surprisingly, the exact replica was so well known and so trivial that it was overlooked by decades. Here Leontieva and Blagosklonny describe the mammalian cellular model: a neglected flask with overgrown cancer cells that turn medium “yellow” (due to lactate accumulation). Such flasks left and forgotten over weekend could be found in any CO2-incubator. The paper is simultaneously startling and obvious. It is obvious from an everyday experience that highly glycollytic cells can destroy cell culture. But like it was known to most researches (90 years ago) that fungi can destroy bacterial culture, it took a special insight to recognize the potential of this seemingly useless phenomenon. There is an intriguing parallel between penicillin and rapamycin. As described in this issue of *Aging*, mTOR pathway is involved in glycolytic phenotype, causing self-poisoning due to over-production of lactic acid. By decreasing lactate production, rapamycin prevents chronological senescence (CS). CS can be manipulated genetically and pharmacologically. Most importantly, the same agents that suppress geroconversion, organismal aging and cancer also suppress CS.

This study does not break any dogma because there was no dogma as the field did not exist. This paper opens a new filed in both aging and cancer research. Blagosklonny and collaborators are currently preparing follow-up papers with special emphasis to cancer research: the ability of rapamycin to decrease lactate production independently of respiration, selection of highly glycolytic cancer cell clones, tumor progression and resistance to therapy. Many questions will be answered. But this first paper defines the field, providing description of the phenomenon, its mechanism and the ways of pharmacological manipulation. It illuminates the place of yeast CS in aging research and its indirect (via common signaling pathways) relevance to cancer and organismal aging. It also rules out altruistic (programmed) aging of yeast because no one would suspect altruistic nature of cancer cells. I invite the readers to enjoy this first paper in the filed of mammalian cell chronological senescence.
